# Insights on CTC Biology and Clinical Impact Emerging from Advances in Capture Technology

**DOI:** 10.3390/cells8060553

**Published:** 2019-06-06

**Authors:** Patrick C. Bailey, Stuart S. Martin

**Affiliations:** 1 Marlene and Stewart Greenebaum Comprehensive Cancer Center, School of Medicine (UMGCCC), University of Maryland, Baltimore, MD 21201, USA; pcbailey@umaryland.edu; 2 Department of Physiology, School of Medicine, University of Maryland, Baltimore, MD 21201, USA

**Keywords:** circulating tumor cells, CTC, liquid biopsy, CTM, CTMat, CTC biology, CTC capture technology

## Abstract

Circulating tumor cells (CTCs) and circulating tumor microemboli (CTM) have been shown to correlate negatively with patient survival. Actual CTC counts before and after treatment can be used to aid in the prognosis of patient outcomes. The presence of circulating tumor materials (CTMat) can advertise the presence of metastasis before clinical presentation, enabling the early detection of relapse. Importantly, emerging evidence is indicating that cancer treatments can actually increase the incidence of CTCs and metastasis in pre-clinical models. Subsequently, the study of CTCs, their biology and function are of vital importance. Emerging technologies for the capture of CTC/CTMs and CTMat are elucidating vitally important biological and functional information that can lead to important alterations in how therapies are administered. This paves the way for the development of a “liquid biopsy” where treatment decisions can be informed by information gleaned from tumor cells and tumor cell debris in the blood.

## 1. Introduction

Cancer remains a leading cause of death in all areas of the world [1]. The primary cause of death however, is not the primary tumor but metastases. The complete biology of metastasis remains unclear, but several general processes are recognized. The initial steps are understood to include the local invasion of the tumor into neighboring tissues followed by intravasation into the circulation, involving either the epithelial to mesenchymal transition (EMT) or the physical shedding of tumor cells into leaky, poorly formed vessels. Both EMT and shedding lead to the dissemination of tumor cells into the lymphatic and hematogenic systems [2]. Of these two methods, hematogenous spread is the most lethal.

Integral to the process of dissemination is circulation in the vasculature. Detached cells are termed circulating tumor cells (CTCs) or, in the case of cell clusters, circulating tumor microemboli (CTM). These cells circulate until they either attach to the vessel endothelium or become lodged in small capillaries. From this point, there can either be migration through the tissue or, in the case of CTMs, possible vascular rupture [3]. Cells which have survived these processes can serve as the seeds of eventual metastatic recurrence.

It has been estimated that tumor cells shed from the primary tumor at a rate of 3.2 × 10^6^ cells per gram of tumor tissue per day, but over half quickly perish [4]. What remains is one cell per 10^6–7^ leukocytes [5]. The rarity and importance of these CTCs has led to the development of many technologies designed to enrich for this small population. Among the challenges inherent in isolating CTCs are the methodologies used for characterizing them. The two main methods that have been employed involve cell surface markers and the physical characteristics of the cell [6], both of which have advantages and pitfalls. The intent of this review is not to exhaustively catalog technologies, but to discuss the principles behind several stand-outs, the importance of CTC isolation in general, possible applications in functional studies and the clinical importance of CTCs in view of biology and new ideas in dissemination modality.

## 2. Diagnostic Importance of CTCs

The presence of CTCs in the blood has been proportionally correlated with poor prognosis, and CTMs are even more strongly correlated with patient outcome [7,8]. For a widespread use of CTC/CTM detection as a diagnostic tool, clinical acceptance is critical. The American Society of Clinical Oncology (ASCO), the National Academy of Clinical Biochemistry, the American Association for Clinical Chemistry, and the American Joint Committee on Cancer have all declined to recommend CTC/CTMat assays in the detection, monitoring or staging of cancer until the benefits of the technique are clarified [9,10,11].

The CellSearch system was approved by the FDA in 2004 for the clinical detection of CTCs but there are numerous challenges inherent in the platform. Problems of physics, statistics, translation, preparation time, and the constraint of fixed cells stained for limited biomarkers have led to inconsistent results [12]. These challenges impact results in detection rate, patient positivity, and correlation with prognosis [6,13,14,15]. Discounting phenotypic heterogeneity between CTCs, there are also numerous technical factors involved in these discrepancies, including differences in technique and bias between operators, sample size and lack of a common reference standard, among many others.

Toward a standard protocol that minimizes these issues, two new trends have a great deal of potential. These are the detection of circulating tumor materials (CTMat) and telomerase activity. As previously mentioned, half of the cells shed from the primary tumor die in circulation. Due to many factors, the membranes of these cells are perforated and cellular contents leak into the blood stream [16]. The physical forces in drawing blood are also a contributing factor to the destruction of viable cells, leading to the accumulation of cellular debris. CTMat is usually captured by the same methods outlined below, but where standard capture technologies would overlook these cell fragments as negative, CTMat capture technology can visualize and enumerate them. Using the CellSpotter technology, which can differentiate between intact tumor cells, damaged tumor cells and tumor cell fragments, CTMat was found to comprise the largest subpopulation in 18 blood samples from prostate cancer patients [16]. CTMat has not only been found to correlate well with viable CTC detection in prognostic capacity, but could also potentially provide an avenue for standardization, insofar as CTMat detection can be more easily quantified. It is also less restrictive in the identification of targets and the process of imaging can be automated [17].

In contrast to the release of cell fragments through apoptosis in the blood stream, another component of CTMat, circulating tumor DNA (ctDNA), is believed to stem mainly from cellular death in the solid tumor [18]. Levels of ctDNA have been found to correlate well with primary tumor resection, chemotherapy and metastasis [19,20]. Although the difficulty in producing primers for PCR of ctDNA fragments is not trivial, this process has been shown to discover relapse well before other conventional methods [21,22] Indeed, ctDNA is already being used for treatment response monitoring, the early detection of relapse [23,24] and even therapy decision (e.g., therapies related to the presence of mutant Epidermal Growth Factor Receptor [EGFR]) [25]. ctDNA from viral associated cancer has also been employed to monitor treatment response [26]. To this end, the analysis of ctDNA can be used to monitor therapeutic success. Increases in mutant alleles as a result of therapy resistance have been shown in patients monitored over a period of two years [25,27]. Finally, the FDA has approved the Cobas EGFR Mutation Test v2 as a companion diagnostic for non-small cell lung cancer therapy with Erlotinib. Standard clinical imaging detection involves the visualizing of a tumor mass, which is a process requiring millions of cells. ctDNA can be monitored and relapse discovered well before this timepoint.

Many of the most utilized platforms for the detection of CTCs utilize epithelial markers for identification, such as cytokeratin and EpCAM (epithelial-cell-adhesion-molecule). This can provide information as to cellular origin but neglects biological behavior. It has also been reported that tumor cells can downregulate or completely lose expression of these epithelial markers during the process of migration and/or dissemination [28]. This creates difficulty for epithelial-based isolations due to their reliance on the EpCAM surface marker for their capture technology. Telomerase, however, has been found to be re-activated in most cancers including prostate, ovarian, breast, lung, colon and bladder [29,30,31,32]. Telomerase activity is also associated with malignancy, is often detected in stage IV cancers and is a marker of stem cell activity [33]. Despite the requirement of lysing the sample for assay preparation, the above factors make this enzymatic activity an attractive choice to detect circulating tumor cells for diagnosis. Especially appealing is the possible application of this assay in the detection of relapse. Basal telomerase activity levels due to T-cell activity and other factors could be established and significant variations from this (apart from infections) could indicate possible tumor relapse.

**Figure 1:**
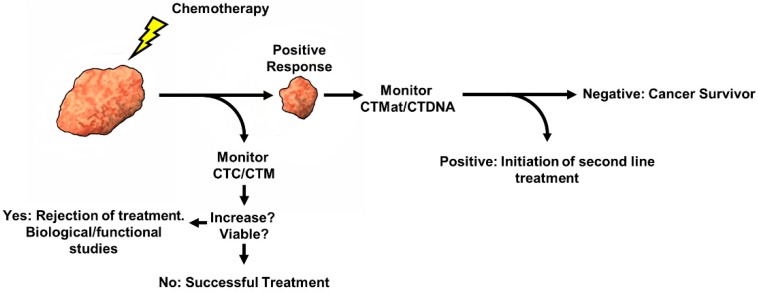
Workflow concept for the analysis of therapy and the early detection of relapse. After chemotherapy, patient CTCs can be analyzed for viability. An increase in viable CTCs can indicate increased mobilization and possible increased risk of relapse. After successful treatment, monitoring patient blood for telomerase activity or ctDNA can give a clinician a much earlier indication of relapse.

Subsequent increases in activity could also reduce the occurrence of false positives. A possible second step to this process that would circumvent the establishment of basal activity would be to negatively select (as outlined below) leukocytes from the sample. If used in combination with monitoring ctDNA, this could be a powerful tool for treating relapse much earlier than currently possible (Figure 1).

## 3. Clinical Relevance

The mobilization of tumor cells into the circulation is integral to distal metastasis. Current thought is that treatment failure due to metastasis is caused by micrometastasis present at the time of treatment or residual local disease [34]. However, there is mounting evidence that treatment methods themselves could cause an increased dissemination of cells into the vasculature or even the activation of dormant metastatic sites [35,36,37,38,39,40,41]. As outlined below, surgery, radiotherapy and systemic chemotherapy can alter tumor biology and possibly influence the risk of metastasis in unforeseen ways. The increase in CTCs as a side effect of treatment is a consideration that deserves careful study. 

The effect of radiotherapy on metastasis has long been studied. Early studies indicated that lower doses of radiation resulted in higher rates of metastasis. Breast cancers transplanted into mice and subjected to non-curative doses of radiation had a 43.5% rate of metastasis compared to 9.6% in the control [42]. Metastasis rates were also 10% higher in transplanted mammary tumors given radiation in addition to resection compared to surgery alone [43]. In experiments with lung cancer and fibrosarcoma, it was shown that irradiated mice had higher rates of distal recurrence compared to control. This was initially explained by the activation of dormant micrometastasis and the modification of local tumor cells into a more aggressive and invasive phenotype [44].

Typical regimens of radiotherapy involve fractionated low doses over the course of many days. After longer periods, tumor cells have typically lost reproductive capacity with successful treatment. However, during the early course of the therapy, tumor cells are much more likely to repair therapy-induced DNA damage [45]. These cells have a higher probability of survival if disseminated into the blood stream. This can be the result of surrounding tissue damage as well as the increased plasticity and genomic instability of irradiated cells [46]. Radiation-induced hypoxia was reported to upregulate the expression of surface markers that increased invasiveness [47]. An increased expression of Vascular Endothelial Growth Factor (VEGF) has also been observed following treatment [48].

The importance of radiation as a therapy cannot be understated. Its clinical value has been demonstrated in many settings. Nevertheless, it has been recently reported that radiation therapy on Non-Small Cell Lung Carcinoma (NSCLC) can mobilize CTCs into the blood stream early in therapy [49]. CTC counts were highest after the first doses of radiation and were shown to originate from the primary tumor. These cells were shown to have increased growth capacity in culture compared to CTCs collected pre-treatment. They also had increased mesenchymal characteristics and were more often found in clusters [8].

Not only radiation, but surgical procedures and chemotherapy have been linked to increased CTCs. Both needle and incisional biopsies have been correlated with increased CTC counts [50,51]. Tumors have also been reported to have formed along the track left by the biopsy needle [52]. Survival rates and local dissemination have been found to be worse with pre-operative biopsies in colorectal cancer, and increased CTCs compared to baseline have also been found both during and after surgery as well [53]. Karigiannis and colleagues have recently reported that neoadjuvant paclitaxel increases both CTCs and metastasis in an MMTV-PyMT (mouse mammary tumor virus-polyoma middle tumor-antigen) murine model [38]. After harvesting the lungs of mice treated with neoadjuvant paclitaxel, they found an increase in both the number and incidence of micrometastasis as well as the presence of single metastatic cells. There was also a twofold increase in CTCs in all experimental models examined, which included xenotransplanted cell lines, the spontaneous PyMT transgenic model and patient-derived xenografts (PDX) [38]. The interrelation between therapy, CTCs and metastasis underscores the vital need to understand the biology of rare circulating cells with the goal of developing targeted treatments. If conventional therapies can potentially increase CTC count and conversely metastasis in some cases, then combination treatments targeting CTCs can potentially improve outcomes.

## 4. Isolation of Cells

The importance of CTCs in diagnosis, prognosis and therapy outcome seems to be clear. Several technologies have been developed for their capture and enumeration. The assays involving ctDNA and CTMat are exciting prospects in the monitoring of recurrence, but neither involve the capture of CTCs for further analysis. Problematically, even with whole cell capture, many techniques kill the cell along the way. Even the FDA-approved gold standard of CTC detection, the CellSearch system, involves chemical fixation. This process is lethal to cells and does not allow for further characterization of viable cells or expansion in culture. Many of the technologies reported in table 1 involve chemical fixation. This does not preclude the modification of the platform’s protocol such that live cells may be captured, but what is commonly reported is outlined in Table 1. In contrast to this, there are many established and developing technologies that have proven to be more sensitive than the CellSearch system and are also designed to capture viable cells, allowing for further biological study [6].

There are several competing modalities in CTC capture methodology, but all of them fall under two conceptual umbrellas: label-based and label-free. Label-based (or affinity-based) capture is the most widely used strategy, with CellSearch as the only technology approved by the US Food and Drug Administration. The prevailing idea behind this methodology is that tumor cells display different surface markers than blood cells and can therefore be separated from the rest of the circulatory cells on this basis. The three most commonly employed biomarkers utilized for tumor cell selection and identification are the epithelial-cell-adhesion-molecule (EpCAM), cytokeratins, and the antigen CD45 [96]. EpCAM is used to positively select for CTCs, while CD45 negatively depletes white blood cells and cytokeratins are used to positively identify CTCs post-enrichment. These three biomarkers have been expanded upon in some technologies in the use of antibody cocktails including, for example, the human epidermal growth factor 2 (HER2) for breast cancer and the prostate-specific membrane antigen (PSMA) for prostate cancer. In most cases, magnetic beads are conjugated to the antibodies allowing for a magnetic field to capture the cell after the antibody binds to its target. Capture strategies also include microfluidic devices with surface-coated antibodies. Cells of interest bind to these antibodies as the sample flows over the surface. Unfortunately, due to the complexity of CTC biomarker expression, there is no single antigen which allows for 100% error-free capture. This makes effective capture a continuing challenge. Table 1 outlines a variety of capture technologies that fall under the umbrellas of “label-based” and “label-free”. Platforms are further characterized by their enrichment principle and their reported capture of live cells.

The CellSearch and Adnatest platforms both make use of magnetic beads attached to antibodies to EpCAM, but Adnatest employs additional cancer-specific antibodies depending on the requirement. CellSearch uses downstream immunostaining to identify CTCs. Positive ID is dependent on the expression of cytokeratins, negative expression of CD45 and positive DAPI nuclear stain. The Adnatest further differs from CellSearch in that it does not rely on downstream immunostaining. Instead, it employs cell lysis and RT-PCR to measure tumor-associated gene expression. A limitation of these technologies is a reliance on EpCAM. EpCAM expression has been shown to vary widely, and cells with low or negative expression can be missed by these platforms [96,97,98,99,100]. Cytokeratin expression can also be lost following EMT [101]. A further drawback is that neither of these technologies allows for further live-cell phenotypic analysis as the captured cells are either fixed or lysed.

Several technologies have been formulated that bypass the requirement for fixation or lysis. Recent advances in microfabrication have allowed the creation of devices with features smaller than a cell. With controlled use of the properties of fluid, cellular contact with these microstructures can be directed. The first among these devices to be developed utilized arrays of antibody-coated microposts [55]. In these devices, sample blood is passed over the chip allowing for the capture of marker-expressing cells. Although some require the pre-lysis of red cells, many enable the use of whole blood with no pre-preparation. The accompanying drawback is that flow rates are most often quite slow at @1–2 mL/h [55,56,102]. The most commonly employed antibody is EpCAM, but several devices employ a cocktail of antibodies that can be specialized for the particular cancer being studied. Today, there are many devices available including the CTC chip, nanopillar chip, micropillar chip, GEDI (geometrically enhanced differential immunocapture) chip, and the OncoCEE among others. These devices have all shown higher capture efficiency than the CellSearch [6], and have the advantage of smaller size and lower cost than the magnetic benchtop devices.

The CTC-chip’s first iteration (preceding the herringbone chip) captured a median of 155 cells/mL in each of 55 samples tested from 68 patients with non-small cell lung cancer, while the CellSearch only captured cells in 20% of patient samples and had a mean of <6 cells/mL [103]. The GEDI chip employs hydrodynamic chromatography by offsetting the microposts in such a way as to separate cells by size and minimize non-specific leucocyte adhesion [56]. The OncoCEE employs a customizable cocktail that can include antibodies for both cancer and mesenchymal specific markers. It also allows for in situ fluorescent staining of the captured cells by staining the capture antibodies [57].

To increase imaging and production efficiency, the field has begun to explore the idea of surface-capture devices that eschew the concept of posts altogether. Microchannels and surface patterns are designed to maximize mixing and surface contact with cells. The simpler design allows for larger scale production and with opaque posts and three-dimensional structure removed, imaging is enhanced. Another welcome enhancement is the allowance of higher flow rates, leading to more rapid throughput [60,62,63]. Devices which use this technology include the microvortex herringbone chip, sinusoidal chip, GEM chip, and the graphene oxide chip.

Biomarkers may also be used to negatively enrich samples containing CTCs. Blood cell markers such as CD45 and/or CD66 can be used to deplete white blood cells from the larger population enriching for CTCs in the remainder. Technologies utilizing this method include EasySep and RosetteSep. RosetteSep incorporates the additional step of density centrifugation, while EasySep uses a magnetic field. A pitfall inherent in this technique is the fact that not all cells in the blood express these markers, resulting in a much poorer purity than with positive selection [74,104,105]. Another downfall is possible CTC loss being caught up in the large movement of concentrated blood cells during depletion. For these reasons, this technique is often used as a preparatory step for other enrichment methods [106].

Despite the utility and many benefits of cellular biomarkers, there are drawbacks as well. It is becoming established that tumor cells express EpCAM at varying levels. In fact, expression can be ablated entirely in some sub-populations, including those which have undergone EMT [107]. Tumor cells have also been reported to express the white blood cell marker CD45 [108]. With these problems in mind, alternative assays which employ only the biophysical properties of the cell have been developed.

These label-free physical detection methods include cell size, deformability, density and electric charge. The most widely employed biophysical selection criterion is cellular size [12]. Tumor cells are larger on average than blood cells [109], and this morphological difference is employed to differentially capture CTCs and CTMs. There are multiple platforms which use these properties such as the micro double spiral chip, the Parsortix and Vortex systems, the micro crescent chip, the Cellsee system, micro column wall chip, ISET, Clear Cell FX, cluster chip, micro pinching chip and the CellSieve among others. Each of these assays have proven to be more selective than the CellSearch system in isolating tumor cells [6].

There are different ways of using size in the process of selection, however. Two-dimensional microfiltration involves a single membrane with variable pore size used to filter out smaller cells while leaving the larger CTCs trapped on the membrane. Cell pore sizes come in a variety of sizes ranging from 6 to 9 um. CellSieve filtration has not only been used to detect cancer-associated macrophages and cancer-associated macrophage-like cells, [110,111] but, using 7.5 mL patient samples, it detected CTCs in 100% of metastatic breast cancer patients tested [88]. CellSieve, ISET and ScreenCell use this methodology, but require pre-processing of the patient sample. FMSA (Flexible Microspring Array) can use whole blood and has been validated in the detection of CTCs in 76% of samples tested in various cancers [112].

**Figure 2:**
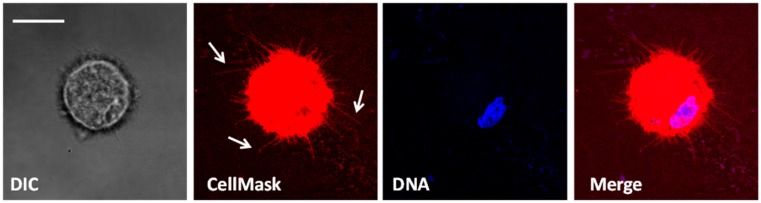
Live CTCs isolated with Parsortix technology. Whole blood was taken from a stage IV metastatic breast cancer patient. The Angle Parsortix was used to isolate CTCs from the blood (15 CTCs in 10 mL). CTCs were tethered to proprietary PEM+Lipid slides and stained with CellMask membrane dye (red). Cells are CD45^-^ and contain a nucleus (blue). Arrows indicate microtubule-based structures termed Microtentacles (McTN).

Three-dimensional filtration systems exploit the larger size of tumor cells, but use multiple layers of filter to capture them. The FaCTChecker, Parsortix system, and cluster chip fall into this category. The FaCTChecker takes advantage of multiple vertical layers with different sized pores [113], while the Parsortix has developed a horizontal stair-type scheme that reduces the channel width stepwise [80]. Viable CTCs can be harvested using either platform. Our lab has employed the Parsortix system to isolate CTCs from breast cancer patients. We subsequently tethered these live cells on a proprietary PEM+Lipid technology [114] and imaged them for Microtentacles (Figure 2). The Cluster Chip is unique in size selection technologies, as its sole target are CTMs. Many technologies have reported on the capture of CTMs, but this novel approach enriches for them specifically while allowing single CTCs to pass through [89]. The design involves staggered rows of triangular pillars. The repeating unit of the design is the cluster trap. This three-triangle arrangement is reminiscent of a biohazard sign insofar as two triangles side by side to create a tunnel that is bifurcated by the third triangle beneath them. This simple design can capture CTMs as small as two cells. The utility of the device was shown in breast, melanoma and prostate cancers, isolating clusters in 41%, 30% and 31% of patients, respectively [89]. Large downsides to filtration systems exist, however. Despite the capture of viable cells without labels that are difficult to remove, the systems are prone to clogging and parallel processing is needed for large volumes. Purity is also an issue as it can range below 10%.

Two exciting new technologies to recently emerge involve the use of inertial fluid forces to passively separate CTCs from the rest of the blood population based on cell size. A combination of shear gradient and wall lift forces interact to stably trap the CTCs. The Vortex platform capitalizes on these forces to inertially focus trapped CTCs in micro vortices created in reservoirs apart from the main fluid channel. Smaller blood cells simply flow by in the main stream. CTCs remain in the device until a slower flow rate flushes them out of the reservoirs. The Vortex Chip processes the standard 7.5 mL sample size in 20 minutes using whole unprocessed blood. Confirmation has come in breast and lung cancers with a purity of 57–94%, much higher than that normally attained with size-based techniques [90]. The ClearCell FX uses inertial forces in combination with secondary flow arising from curved channels [115]. When a channel is curved, there is a difference in the flow rates between the center of the channel and the walls. This difference in flow rates is termed a “Dean’s” flow and, when combined with inertial forces, can be calculated to precisely position cells. The trapezoidal channel results in larger cells on the shorter wall and smaller cells on the larger wall. This channel then splits into two collection outlets, where CTCs are isolated and captured. This technology requires red cell lysis prior to flow but has an impressive 8-minute run time. It has been confirmed in breast and lung cancers with a higher capture rate than the Vortex [116]. Both processes involve minimal stress on cells without the use of labels and are much simpler to fabricate than those previously mentioned.

Dielectrophoresis (DEP) exploits the electrical characteristics of tumor cells. These characteristics depend on phenotype, composition and morphology. DEP polarizes cells by using a nonuniform electric field. This results in the ability to physically manipulate the cells by exerting attractive or repulsive forces (positive pDEP or negative nDEP). ApoStream employs a strategy wherein the electrical field separates tumor cells and leukocytes, using differences in their conductivity. The field attracts CTCs and repels leukocytes. After pre-processing by centrifugation, the ApoStream can process captured CTCs from 10mL of whole blood in less than an hour [117]. 

The DEPArray applies the second DEP strategy, retention, by trapping single cells in DEP cages generated via an array of individually controllable electrodes [118]. DEPArray as a platform is not designed for the bulk enrichment of cells, however. It is intended for single cell capture. Multiple studies have shown the utility of the technology in this capacity [95,119,120], but an unfortunate drawback is large cell loss during sample preparation [121].

## 5. CTC Biology

The prognostic importance of CTC counts is well established, but counts have not yet been widely employed to affect clinical decisions, due to unclear relevance to treatment. CTC counts have therefore not been recommended clinically to affect treatment decisions, as of yet [122]. Consequently, a more robust understanding of CTC biology is required. Tumor heterogeneity is increasingly being reported in the literature, not only between primary and secondary tumors, but intratumor as well. There can be as many as six different clonal cell lines within just one tumor [123]. Standard biopsy techniques such as fine needle aspiration and core biopsy are insufficient to capture this variety. These techniques, by design, take tissue from one area of the tumor for further analysis. Even with multiple samples, such as those taken in prostate cancers, there is not sufficient tissue to encompass all of the heterogeneity. “Liquid biopsy” is a term being increasingly used to describe analysis of CTC populations. The CTC population is thought to encompass more of the clonal populations in a tumor [122]. By analyzing the captured cells, an investigator can get a more complete picture of tumor composition and how it changes over time.

**Figure 3:**
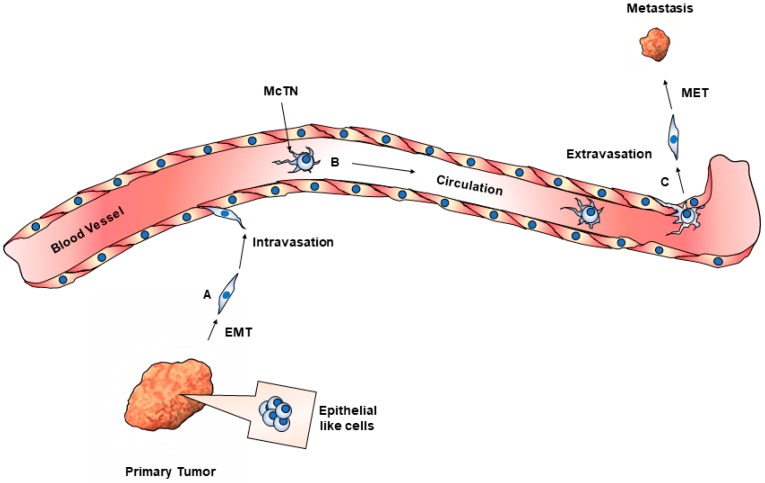
Epithelial to Mesenchymal Transition (EMT) and metastasis. (**A**) Epithelial-like cells in the primary tumor undergo a transition to a mesenchymal phenotype and migrate towards the vasculature. (**B**) Detached tumor cells in the circulatory vessels display microtubule-based structures, termed Microtentacles (McTN). (**C**) McTN aid in reattachment and extravasation. Extravasated cells undergo a mesenchymal to epithelial transition, and seed tumors at distal sites.

Studies of the composition of CTCs can further shed light into the process of metastasis. The complete process of metastasis is unclear, but conventional wisdom describes a process where tumor cells undergo the epithelial mesenchymal transition (EMT). This process involves cells detaching from the main tumor body, migrating through the extracellular matrix and extravasating into the circulation (Figure 3). During this process, the cell downregulates the expression of its epithelial markers, such as E-cadherin, and upregulates EMT markers, such as N-cadherin, snail, twist, vimentin and detyrosinated tubulin [123].) CTC/CTMs have been shown to upregulate vimentin and detyrosinated tubulin as well [124]. After extravasation, the cell then undergoes the reverse process of mesenchymal-to-epithelial transition (MET). This has been widely held to be the main mode of metastatic dissemination, but new reports have begun to challenge this.

Fischer and colleagues described an experiment with a triple transgenic mouse that tracked mesenchymal lineage in breast cancer tissue. The system utilized an irreversible color switch that was activated by the expression of fsp1, a crucial protein in EMT initiation. With the expression of fsp1, cells experiencing EMT would undergo an irreversible color change from red to green, allowing for the tracking of any metastatic cell that had gone through the process. What was observed was that the vast majority of metastatic tumor tissue was red and had not undergone EMT. This was confirmed using multiple oncogenes and EMT tracing proteins. Interestingly, the following chemotherapy tumor recurrence was mostly green [125]. Similar findings were reported independently in the same issue of *Nature*, from a lab using twist and snail in pancreatic tumor lines [126].

The ramifications of these findings are manifold and beyond the scope of this review to cover. It is however important to note that this is a proof-of-principle that the process of EMT can be dispensable for initial metastasis, in some cases. This underscores the importance of understanding the biology in circulating cells. Which proteins CTCs express, and the resulting phenotypes, are crucial to understanding how cancer spreads to distal sites. It is indeed possible that the bulk of tumor spread results from simple CTC shedding into the vasculature. This does not reduce the importance of EMT in cancer, however. Cancer cells displaying the mesenchymal phenotype have been shown to be more aggressive, stem-like, and resistant to treatment [127]. Both Zheng and Fischer also observed EMT cells persisting after treatment despite original metastasis composition. What this highlights is that there can be multiple modes of metastasis, and the study of cells in transition can give us insights into the process.

Aceto et al. have recently shown that CTMs are 23–50 times more metastatic than CTCs [8]. Their use of fluorescently labeled cells also highlighted that clusters arise from oligoclonal groupings of cells that differentially express the cell junction protein plakoglobin. These studies, along with the results of Zheng and Fischer, further emphasize the importance of circulating cell study. They give us insight into the probable mechanism of metastasis. In the 323 lung foci that Aceto observed, 171 were CTM-derived, although CTMs only comprised 2–5% of the total population of tumor cells in the circulation.

Previous thought was that CTMs were likely to break up in the physical pressures of the blood stream, or to become lodged very quickly in smaller capillaries, negating their capability of seeding distant metastasis [128]. Recent work has shown this is not the case. Au et al. demonstrated with microscopy and capillary tubing that tumor clusters migrated in a single file fashion without dissociation. Moreover, the clusters were viable upon capillary exit [129]. Taken together with the evidence that clusters have a much higher metastatic potential, the benefit of elucidating biological differences between CTCs and CTMs is clear. In fact, very recent evidence has indicated that the disruption of CTMs leads to the suppression of metastasis [130].

It has been hypothesized that CTMs could arise either by passive shedding or through collective migration [101,131]. Collective migration has been observed in multiple tumor types, but it has only been directly correlated to local invasion [101]. Metastasis, through collective migration, has merely been inferred by the presence of clusters in the blood. Tumor vasculature is improperly formed, tortuous, leaky, and possessive of blind shunts [132]. It has been reported that tumor cells can actually replace vascular endothelium in places, a process known as vasculogenic mimicry [133]. With these factors in mind, it is quite feasible that CTCs and CTMs mainly arise through the passive sloughing of cells. This would correlate well with the data showing that breast cancers arising from *neu* and PyMT transgenes undergo very little EMT.

Interstitial fluid pressure (IFP) could contribute to CTC shedding as well. IFP is the fluid pressure measured within tumors and is the direct result of hyperpermeable blood vessels. Fluid and plasma proteins extravasate into the tumor tissue and elevate the pressure in the interstitium [47]. Not only could this increased pressure disrupt cell–cell junctions, but it could cause physical pressures that assist in cells detaching from the tumor bulk. High IFP is correlated strongly with poor prognosis [134]. As higher interstitial pressure is a direct result of improperly formed vessels, and stronger pressure could result in cell detachment, it follows that cells could break off at a higher rate as capillaries become leakier.

## 6. Functional CTC Studies

**Figure 4:**
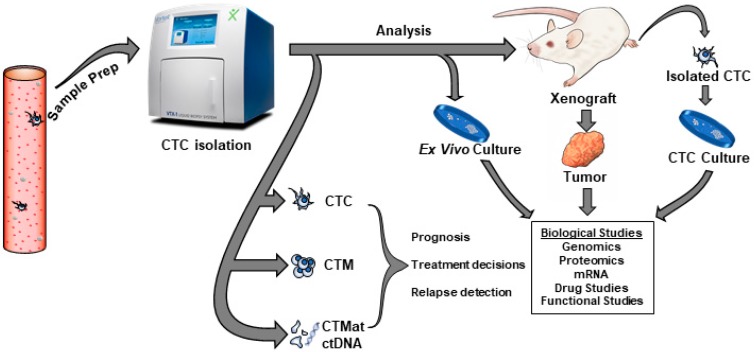
Workflow concept for the isolation of CTCs and subsequent analysis. Patient blood is passed through a capture device which enriches for tumor cells. Captured cells are then identified, enumerated and characterized. Cells can then be cultured and subjected to further biological and functional analysis.

Translating lab research into clinical practice involves the study of how cells function, both in vitro and in vivo. As outlined above, it has been clearly shown that higher CTC counts in peripheral blood correlates with poor prognosis. Functional studies can broaden the spectrum of applications to CTC analyses. The challenges in obtaining stable cultures are significant but advances in CTC expansion from patient samples have been achieved. The subsequent functional studies can give clues into the identity of metastasis-initiating cells and can point the way to new avenues of therapy. A workflow, as outlined in Figure 4, illustrates the concept of CTC study, beginning with isolation and ending with the functional study of cultured CTCs. The first step in a workflow of this kind would be sample preparation and isolation using one of the methods outlined above. This would result in the capture of differing circulating materials, depending on the capture technology. These captured materials could eventually be used for prognosis and relapse decisions.

Functional analysis of CTCs has been performed in multiple studies. Zhang et al. reported a protocol for the primary culture of breast cancer CTCs from patients with advanced stage and brain metastases [135]. The cultures survived for several weeks. This study allowed the elucidation of several biomarkers, including HER2 and EGFR, as brain metastasis selected markers (BMSM). Cells which expressed this BMSM signature exhibited significant invasiveness and resulted in brain metastases in murine xenografts. Oligoclonal breast cancer CTC cell lines were cultured for >6 months in 2014 [136]. Of five tested lines, three proved to be tumorigenic. The culture allowed for the discovery of new mutations in the estrogen receptor gene, fibroblast growth factor and PIK3CA. A long term culture of a CTC line from prostate cancer was also established using a novel 3D organoid system [137]. This included TRMPRSS2-ERG fusion proteins, overexpression of SPINK1 and SPOP and CHD1 mutations and loss, respectively. Lung cancer CTCs were successfully expanded ex vivo using a 3D co-culture which used a simulated tumor microenvironment. CTCs expanded from 14/19 patient samples and had matched mutations with their respective primary tumors, including tp53 [138].

Captured breast cancer CTCs were injected into murine tibia bone resulting in lung, liver and bone metastases [104]. The study of protein expression in the metastasis revealed universal expression of EpCAM, MET, CD44 and CD47. This could reveal important information on necessary proteins in the process of engraftment and metastatic outgrowth. Further study in an additional cohort revealed that metastases increased with the number of CD44/CD47/MET/EpCAM-positive cells. Importantly, these cells were obtained from advanced stage patients with high numbers of CTCs. This underscores the need to obtain and expand tumor cells from early stage patients to confirm this protein expression profile as metastasis-initiating in all stages.

Migratory capabilities of isolated metastatic prostate CTCs were shown in NOD/SCID mice [139]. Tumor cells were found in the spleen and the bone marrow after xenografting. Hodgkinson et al. showed that CTC xenografts of small cell lung cancer (SCLC) are not only tumorigenic in murine models but respond similarly to chemotherapy as in the original donor patient. SCLC patients have been reported to have the highest CTC counts of all solid tumors [140]. Notably, these tumors are often inoperable and difficult to biopsy. Expanding tumors which mirror patient response is an important step in furthering treatment less invasively.

## 7. Conclusions

Metastasis remains the number one cause of death in cancer patients. This is the result of the migration of cells from the primary tumor to distal sites. Indispensable to this process is the migration/shedding of CTCs into the vasculature. These circulating tumor cells can be analyzed for a breadth of beneficial information. Currently, prognostic indications can be made based on the enumeration of CTCs in the blood. With further technological development, the presence of metastasis could be detected before clinical manifestation, by monitoring tumor materials in the blood. It is also feasible that patients with known genetic risk factors could be monitored for ctDNA, using primers for known tumor mutations. This could possibly advance diagnosis by years, and increase survival rates significantly.

Even after disease control is accomplished with surgery and/or therapy, metastasis can remain a problem. This can be partially due to cancer cell mobilization caused by therapy itself. Radiation has been shown to select for and to convert tumor cells to phenotypes that are more mobile and aggressive, allowing for the generation of metastases. Tissue disruption and the leakage of blood containing tumor cells during surgery can also promote tumor spread. This includes procedures such as routine biopsy.

These problems underscore the need for the capture and study of viable tumor cells. Many technologies exist, but many involve the fixation of cells and their subsequent death. Emerging platforms have developed ways to isolate live CTCs which allow for downstream biological analysis. These studies have led to valuable insights into the mechanisms of metastasis and cellular survival in the harsh environment of the circulation. Functional studies with cultured CTCs and xenografts have revealed important information on protein expression and genetic composition. With the standardization of capture techniques, inconsistencies in efficiency can be greatly reduced, allowing for more robust information to be attained.

All these principles could support the goal of improving drug discovery to reduce metastasis. The current cancer detection and drug treatment paradigm involves tumor growth and visualization. Current technological parameters limit the tumors we can visualize to upwards of ten million cells. A shift of focus to the detection of ctDNA/CTMat/CTC/CTMs can improve detection sensitivity and improve treatment strategies. If surgery and radiation can promote cellular dissemination, then therapies that specifically target circulating cells could increase survival outcomes and reduce distal recurrence. Overall, developing therapies that target cancer’s ability to ever survive in circulation can prevent metastasis before it occurs.

## Figures and Tables

**Table 1:** Circulating tumor cell (CTC) technologies. CTC isolation technologies grouped by category and isolation criteria. Modified from Ferreira et al. 2016 [54]. *refers to the reference in question.

Subcategory	Platform	Enrichment Principle	Live Cell Analysis Reported *	Company
**Label-Based**				
Positive Enrichment Immunoaffinity				
Micropost Arrays	CTC-Chip [55]	EpCAM	Yes	
	GEDI Chip [56]	PSMA/HER2, Size	No	
	OncoCEE [57]	Antibody Cocktail	No	Biocept Inc. San Diego, CA, USA
Microfluidic Surface Capture	Biofluidica CTC system [58]	EpCAM	Yes	Biofluidica Inc.San Diego, CA, USA
	CytoTrapNano [59]	EpCAM	No	Cytolumina. Los Angeles, CA, USA
	GEM Chip [60]	EpCAM	Yes	
	HTMSU [61]	EpCAM	No	
	Graphene Oxide Chip [62]	EpCAM	No	
	Herringbone Chip [63]	EpCAM	No	
Microfluidic Magnetic	Ephesia [64]	EpCAM	Yes	
	Magnetic Sifter [60]	EpCAM	No	
	LiquidBiopsy [65]	Antibody Cocktail	No	Thermo Fisher, Waltham, MA, USA
	Isoflux [66]	EpCAM	No	Fluxion Biosciences, Alameda, CA, USA
Magnetic	CellSearch [67]	EpCAM	No	Silicon Biosystems, Huntington Valley, PA, USA
	AdnaTest [68]	Antibody Cocktail	No	Qiagen, Hilden, Germany
	MACS [69]	EpCAM	No	Miltenyi Biotec, Bergisch Gladbach, North Rhine-Westphalia, Germany
	MagSweeper [70]	EpCAM	No	
Magnetic in vivo	CellCollector [71]	EpCAM	Yes	GILUPI, Potsdam, Germany
Negative Enrichment Immunoaffinity				
Magnetic	EasySep [72]	CD45	No	STEMCELL, Vancouver, BC, Canada
	QMS [73]		Yes	
	MACS [74]		Yes	Miltenyi Biotec, Bergisch Gladbach, North Rhine-Westphalia, Germany
Microfluidic/Magnetic	CTC-iChip [75]	CD45, CD66b, Size	Yes	
**Label-Free**				
Density				
	Ficoll-Paque [76]	Density	Yes	GE Healthcare Bio-Sciences, Pittsburg, PA, USA
	OncoQuick [77]	Density, Size	Yes	Greiner Bio-One, Kremsmünster, Austria
	RosetteSep [78]	Density, Antibody Cocktail	Yes	STEMCELL, Vancouver, BC, Canada
	Accucyte and CyteSealer [79]	Density	Yes	Rarecyte, Seattle, WA, USA
Size				
Filtration	Parsortix [80]	Size, Deformability	Yes	Angle, King of Prussia, PA, USA
	Microwall Chip [81]		Yes	
	ScreenCell [82]		Yes	ScreenCell, Westford, MA, USA
	Resettable Cell Trap [83]		Yes	
	Flexible Micro Spring Array (FMSA) [84]		Yes	
	FaCTchecker [85]		Yes	Circulogix, Hallandale Beach, FL, USA
	Crescent Chip [86]		Yes	
	ISET [87]		Yes	RareCells Diagnostics, Paris Cedes, France
	CellSieve [88]		Yes	Creatv Microtech, Potomac, MD, USA
	Cluster Chip [89]		Yes	
Fluid Dynamics	Vortex [90]	Size	Yes	Vortex Biosciences, Pleasanton, CA, USA
	Double Spiral Chip [91]		Yes	
	Micropinching Chip [92]		Yes	
	ClearCell FX [93]		Yes	Genomax Technologies, Singapore
Electric				
	ApoStream [94]	Electrical Signature	Yes	Apocell, Houston, TX, USA
	DEPArray [95]		Yes	Silicon Biosystems, Huntington Valley, PA, USA
